# PHI-base in 2022: a multi-species phenotype database for Pathogen–Host Interactions

**DOI:** 10.1093/nar/gkab1037

**Published:** 2021-11-12

**Authors:** Martin Urban, Alayne Cuzick, James Seager, Valerie Wood, Kim Rutherford, Shilpa Yagwakote Venkatesh, Jashobanta Sahu, S Vijaylakshmi Iyer, Lokanath Khamari, Nishadi De Silva, Manuel Carbajo Martinez, Helder Pedro, Andrew D Yates, Kim E Hammond-Kosack

**Affiliations:** Department of Biointeractions and Crop Protection, Rothamsted Research, Harpenden AL5 2JQ, UK; Department of Biointeractions and Crop Protection, Rothamsted Research, Harpenden AL5 2JQ, UK; Department of Biointeractions and Crop Protection, Rothamsted Research, Harpenden AL5 2JQ, UK; Department of Biochemistry, University of Cambridge, Cambridge CB2 1GA, UK; Department of Biochemistry, University of Cambridge, Cambridge CB2 1GA, UK; Molecular Connections, Kandala Mansions, Kariappa Road, Basavanagudi, Bengaluru 560 004, India; Molecular Connections, Kandala Mansions, Kariappa Road, Basavanagudi, Bengaluru 560 004, India; Molecular Connections, Kandala Mansions, Kariappa Road, Basavanagudi, Bengaluru 560 004, India; Molecular Connections, Kandala Mansions, Kariappa Road, Basavanagudi, Bengaluru 560 004, India; European Molecular Biology Laboratory, European Bioinformatics Institute, Wellcome Genome Campus, Hinxton, Cambridge CB10 1SD, UK; European Molecular Biology Laboratory, European Bioinformatics Institute, Wellcome Genome Campus, Hinxton, Cambridge CB10 1SD, UK; European Molecular Biology Laboratory, European Bioinformatics Institute, Wellcome Genome Campus, Hinxton, Cambridge CB10 1SD, UK; European Molecular Biology Laboratory, European Bioinformatics Institute, Wellcome Genome Campus, Hinxton, Cambridge CB10 1SD, UK; Department of Biointeractions and Crop Protection, Rothamsted Research, Harpenden AL5 2JQ, UK

## Abstract

Since 2005, the Pathogen–Host Interactions Database (PHI-base) has manually curated experimentally verified pathogenicity, virulence and effector genes from fungal, bacterial and protist pathogens, which infect animal, plant, fish, insect and/or fungal hosts. PHI-base (www.phi-base.org) is devoted to the identification and presentation of phenotype information on pathogenicity and effector genes and their host interactions. Specific gene alterations that did not alter the *in host* interaction phenotype are also presented. PHI-base is invaluable for comparative analyses and for the discovery of candidate targets in medically and agronomically important species for intervention. Version 4.12 (September 2021) contains 4387 references, and provides information on 8411 genes from 279 pathogens, tested on 228 hosts in 18, 190 interactions. This provides a 24% increase in gene content since Version 4.8 (September 2019). Bacterial and fungal pathogens represent the majority of the interaction data, with a 54:46 split of entries, whilst protists, protozoa, nematodes and insects represent 3.6% of entries. Host species consist of approximately 54% plants and 46% others of medical, veterinary and/or environmental importance. PHI-base data is disseminated to UniProtKB, FungiDB and Ensembl Genomes. PHI-base will migrate to a new gene-centric version (version 5.0) in early 2022. This major development is briefly described.

## INTRODUCTION

Infectious diseases are a major concern to the health of plants, animals, humans and to the entire ecosystem. Locally and globally infectious diseases threaten food, feed and fibre security, human community structures, the economic wealth of regions, countries and continents as well as the biodiversity of natural and human-restored aquatic and terrestrial ecosystems (1–4). The increasing effects of human migration and travel, the globalization of the trading of fresh goods and climate change, have resulted in a rise in the incidence and severity of existing diseases, alongside the emergence of many novel pathogen species, new strain variants with enhanced disease-causing abilities, and a rise in zoonotic infections (5). Climate change, and in particular rising global temperature, is causing many pathogenic species to migrate polewards: as a result, plant host species are encountering unfamiliar pathogens and novel disease outbreaks are occurring (6,7). In addition, the range of commercial anti-infective chemicals available to control infectious diseases effectively is gradually diminishing, either because of the emergence or re-emergence of chemical-resistant species or strains, or through a rise in legislation banning or restricting the use of previously registered chemistries (8). As a result, year on year the burden of microbial infections is of growing concern to human, animal and plant health (1,2,5).

During infectious disease formation, a series of complex and dynamic interactions between pathogenic species and their potential hosts occur. These interactions result in the pathogen successfully deploying a suite of virulence factors and secreted effectors that suppress, thwart or minimize the host's ability to recognize and/or respond to the pathogen. The host loses its ability to mount an effective defensive response and as a result, the pathogen succeeds in infecting the host. For obligate biotrophic pathogens, an extra requirement for successful infection is to ensure the colonized host cells remain alive throughout the infection process. Alternatively, during these dynamic interactions, the host's recognition and defensive mechanisms are successfully activated, the deployed pathogen virulence factors and effectors are ineffective, and the host remains disease-free and healthy (9,10). In recent years, it has become increasingly clear that by studying host–pathogen interactions across the tree of life, new underlying biological principles can be uncovered. For example, in plant–pathogen interactions, similar cellular compartments (i.e. chloroplast and nucleus) are now recognized to be targeted by non-homologous small proteinaceous effectors produced by a range of bacteria, fungal and/or protist pathogenic species with different *in vivo* lifestyles (11). Also, many animal and plant infecting pathogens are now known to use molecular mimicry of essential host molecules, either functionally or structurally, to gain the advantage during infection (12,13). As precise gene function studies become possible for an ever-increasing range of pathogenic species, often involving both natural and experimental host species, the knowledge that can be gained from comparative interspecies analyses has grown rapidly. In addition, in the post-genomics era, where the amount of genomic data is doubling every seven months, not only are fully sequenced, assembled and annotated genomes available for thousands of pathogenic species and their hosts, but also an increasing number of pathogen pan-genomes are available for particularly problematic species and species complexes.

With this abundance of new data and new data types, there is growing scientific and commercial interest in omics approaches such as comparative pathogen genomics, comparative host–pathogen genomics, and whole genome protein–protein interaction (PPI) predictions. These methods allow (i) predicting and identifying functionally homologous genes in pathogens and hosts, (ii) identifying species-unique genes and pathways, and (iii) pinpointing sequence variants and gene sequence nulls that lead to alternative interaction outcomes. Collectively, this increased understanding of the dynamic mechanisms and principles controlling a wide range of interactions will contribute to what have traditionally been the two predominant approaches available for combating infectious disease: namely, activating the host immune system to prevent infection, and precise use of commercial anti-infective chemicals to eliminate infectious agents (14–16). These approaches have now been joined by others, including intervention by highly specialized biological control agents (biopesticides) (17), and the use of RNA interference strategies and genome editing to remove or modify pathogen susceptibility targets in the host (14).

In 2005, the Pathogen-Host Interactions database (PHI-base) was established and made freely available at www.phi-base.org. PHI-base adheres to the FAIR principles to ensure data is Findable, Accessible, Interoperable, and Reusable (18). In 2016, the project joined the UK node of the European life-sciences infrastructure for biological information (ELIXIR) project, which is focused on providing sustainable bioinformatics resources, as a supplier of agrigenomics data (19) (https://elixiruknode.org). PHI-base stores expertly-curated molecular and biological information on genes proven to affect the phenotypic outcome of pathogen–host interactions (20,21). Each PHI-base entry is supported by strong experimental evidence from a peer-reviewed publication. In PHI-base, the term ‘interaction’ is specifically defined as the observable function of one gene, on one host and on one tissue type (20). PHI-base entries include experimentally verified pathogenicity, virulence, and effector genes from bacterial, fungal and protist pathogens which infect plant, human, animal, insect and other hosts. Also included is information on the first host targets of pathogen effectors and the targets of commercial anti-infective chemicals. Viruses are not included in PHI-base, due to their extensive coverage in other databases. To enhance PHI-base's use for comparative studies, genes tested but found not to affect the interaction outcome are also curated. Nine high-level phenotypic outcome terms have been defined to permit the comparison of interactions across the entire tree of life (22). These terms are ‘loss of pathogenicity’, ‘reduced virulence’, ‘increased virulence (hypervirulence)’, ‘unaffected pathogenicity’, ‘effector’, ‘lethal’, ‘enhanced antagonism’, ‘resistance to chemical’ and ‘sensitivity to chemical’. These terms are particularly useful for biologists and bioinformaticians who are undertaking cross-discipline analyses or mega-scale data analyses and are unfamiliar with the nuances of multiple pathosystems, but who wish to include pathogens with different host ranges, lifestyles and niche occupancies in their comparative analyses. To further increase the utility of PHI-base, particularly to biologists, a BLAST tool (PHIB-BLAST, phi-blast.phi-base.org) is available to permit BLAST queries arising from functional genomics, transcriptomics, proteomics and protein–protein interaction experimentation.

Since 2011, the phenotypic data in PHI-base has been directly connected to the individual gene entries within the genomes of plant pathogenic species available within Ensembl Fungi, Ensembl Bacteria and Ensembl Protists (23,24). More recently, PHI-base phenotype annotations have also been displayed within FungiDB (25). PHI-base also reuses ontologies and resources provided by external resources, including PubMed, NCBI Taxonomy (26), UniProtKB (27), the Gene Ontology (GO) (28), ChEBI (29) and FRAC (www.frac.info). Several complementary multi-species databases on pathogens exist that also provide gene function annotation (reviewed by (20,30,31)). The newest multispecies plant pathogen database, SecretEPDB, focuses on cataloguing knowledge on the effectors produced by various animal or plant infecting bacteria (32). PHI-base remains unique in describing a wide range of plant, human, animal and insect pathogen–host interactions using the same controlled generic vocabulary consistently across more than 270 species.

In this article, we report on a major increase in PHI-base gene content, how pathogen strain and disease names have been amended, links to other data resources and the release of a new gene-centric web interface of the database, PHI-base 5.

## RESULTS AND DISCUSSION

### Biological data

Version 4.12 of PHI-base (released in September 2021 and described in this article), contains 8411 genes, 18190 pathogen–host interactions (PHIs), 279 pathogens, 228 hosts and 4387 references. The number of genes manually curated for PHIs has increased by 24% since version 4.8 (reported in 2020) (21). Bacterial and fungal pathogens provide 96.4% of the PHI phenotype annotations (of which 54% involve bacterial pathogens and 46% involve fungal pathogens), whilst protists, protozoa, nematodes and insects provide 3.6% (Table 1). The Ascomycete fungi dominate the fungal pathogen curation with 7102 PHI phenotype annotations and 103 species (88% of all fungal PHI phenotypes), followed by the Basidiomycetes with 966 PHI phenotypes and 11 species (12% of all fungal PHI phenotypes). Compared to version 4.8, an additional 4391 PHI phenotype annotations describing experimental data for 1842 genes from 932 newly manually curated publications are included up to March 2021.

**Table 1.** Summary of pathogen groups, interactions and phenotypes within PHI-base version 4.12

Data type	Bacterium	Fungus	Protist	Nematode	Insect	Totals
Number of pathogens	141	116	14	5	2	279
Interactions in total	9516	8134	500	26	10	18 186
**PHI phenotypes**						
Loss of pathogenicity	235	737	10	1	0	983
Reduced virulence	4738	3776	140	13	0	8667
Unaffected pathogenicity	2022	2600	68	0	0	4690
Effector (plant avirulence determinant)	1850	523	246	11	10	2640
Increased virulence (hypervirulence)	639	301	28	1	0	969
Lethal	19	156	8	0	0	183
Chemical target: resistance to chemical	7	29	0	0	0	36
Chemical target: sensitivity to chemical	6	8	0	0	0	14
Enhanced antagonism	0	4	0	0	0	4

The number of pathogenic species in PHI-base has increased by 11 to total 279. New species include newly emerging pathogens under intense investigation and species included in comparative studies. Within PHI-base, plant pathogens represent ∼54% of the species investigated (Table 2). There continues to be an almost equal split between cereal and non-cereal infecting species curated in PHI-base. Tree and woody shrub infecting species provide 1316 plant PHI annotations, involving 61 species (13.4% of the plant PHIs), of which 945 PHIs are for economically important fruit-bearing species in the genus *Citrus*, *Malus*, *Prunus* or *Pyrus*. The three model plant species *Arabidopsis thaliana*, *Nicotiana benthamiana*, and *Nicotiana tabacum* continue to provide ∼5% of the data (961 PHIs). Over the past two years, the number of curated PHI phenotypes for pathogens that infect humans and their model hosts has increased to 38% of the total, while 32% of new annotations come from agricultural crop infecting species. This change in PHI curation emphasizes the continuing recent shift to fundamental investigations into human–pathogen and animal–pathogen interactions using surrogate model species. Also, the increasing availability of fully sequenced, assembled and well annotated genomes for pathogens of humans and animals has led to increased interest by a wider range of researchers and hence increased rates of discovery and hypothesis testing. New pathogen species that have been curated for the first time include *Streptococcus mutans*, *Orbilia oligospora* and *Pseudomonas cannabina* (Supplementary Table S1).

**Table 2.** Summary of the number of host species and interactions within PHI-base version 4.12

Data type	Plant	Vertebrate	Insect	Nematode	Others
Host species	141	38	32	3	14
Interactions in total	9845	6712	1090	363	5
**PHI phenotypes** ^†^					
Loss of pathogenicity	676	273	22	11	1
Reduced virulence	3738	4050	601	206	78
Unaffected pathogenicity	2734	1510	316	121	20
Effector (plant avirulence determinant)	2270	336	29	1	5
Increased virulence (hypervirulence)	295	529	120	24	1
Chemical target: resistance to chemical	27	3	0	0	0
Chemical target: sensitivity to chemical	13	1	0	0	0
Enhanced antagonism	4	0	0	0	0

^†^ The ‘Lethal’ high-level phenotype is not included since it is not applicable for host species: this phenotype indicates that a mutation in a pathogen renders the pathogen inviable.

The 30 most annotated pathogen species in PHI-base now account for 72.3% of the total PHI data, which is provided by the curation of 6111 genes (Table 3). Included in the highly annotated species list are six plant pathogenic fungi, five plant pathogenic bacteria, 13 animal pathogenic bacteria, four animal pathogenic fungi, one bacterial species able to infect both plant and animal hosts, and one fungal species able to infect insect hosts. As in previous versions of PHI-base, the highest number of pathogen–host interactions and pathogen genes recorded from the literature are from the filamentous fungal pathogens *Fusarium graminearum* and *Magnaporthe oryzae*, which cause various diseases on staple cereal crops, such as wheat, barley, rice and maize. The most highly represented plant-infecting bacteria are: *Xanthomonas oryzae*, a pathogen of rice; *Ralstonia solanacearum*, a pathogen of potato and other *Solanaceae* species; and various pathovars of *Pseudomonas syringae* which cause disease on different horticulturally important fruit and vegetable crop species. For the animal kingdom, the most curated pathogens include the human pathogens *Salmonella enterica*, *Candida albicans, Cryptococcus neoformans, Escherichia coli* and *Aspergillus fumigatus* (Table 3). Across all species in PHI-base, the number of genes annotated with a phenotype varies greatly, from 59 to 1279, and this reflects not only the size of the research community for the species and the funds available, but also the inherent difficulty of the experimental pathosystem(s).

**Table 3.** Highly annotated pathogens, interactions and proteins within PHI-base version 4.12

Pathogen	Interactions	Proteins*	Loss of pathogenicity	Reduced virulence	Increased virulence	Effector	Unaffected pathogenicity	Lethal	No. of host species
*Fusarium graminearum* ^†^ (F)	1711	1279	40	610	9	0	958	94	14
*Magnaporthe oryzae* ^†^ (F)	1409	643	289	594	16	84	425	1	7
*Salmonella enterica* ^‡^ (B)	1022	487	9	602	71	137	203	0	15
*Candida albicans* ^#^ (F)	641	333	57	393	54	0	133	4	13
*Cryptococcus neoformans* ^‡^ (F)	449	246	52	274	24	0	89	10	10
*Escherichia coli* ^‡^ (B)	504	241	1	302	33	18	149	1	16
*Pseudomonas aeruginosa* ^‡† #^ (B)	592	234	19	300	43	4	226	0	23
*Aspergillus fumigatus* ^‡^ (F)	389	222	33	180	21	0	111	42	7
*Xanthomonas oryzae* ^†^ (B)	595	218	3	138	27	308	119	0	3
*Ustilago maydis* ^†^ (F)	417	206	50	217	9	17	124	0	3
*Staphylococcus aureus* ^‡^ (B)	554	194	12	338	95	2	106	1	14
*Pseudomonas syringae* ^†^ (B)	340	172	1	79	9	198	52	1	16
*Botrytis cinerea* ^†^ (F)	419	131	24	248	14	4	127	0	28
*Ralstonia solanacearum* ^†^ (B)	879	125	16	65	1	784	12	1	12
*Erwinia amylovora* ^†^ (B)	506	122	34	202	55	15	200	0	6
*Fusarium oxysporum* ^‡^ (F)	260	121	25	120	10	30	75	0	22
*Xanthomonas campestris* ^†^ (B)	198	121	11	108	4	40	33	2	8
*Mycobacterium tuberculosis* ^‡^ (B)	173	116	3	86	36	1	47	0	4
*Streptococcus pneumoniae* ^‡^ (B)	185	107	4	123	10	0	42	6	5
*Beauveria bassian*a^§^ (F)	132	90	0	0	0	0	0	0	11
*Klebsiella pneumoniae* ^‡^ (B)	198	85	5	84	4	0	105	0	5
*Vibrio cholerae* ^‡^ (B)	158	78	1	103	5	0	49	0	7
*Streptococcus pyogenes* ^‡^ (B)	181	75	0	112	20	0	47	2	8
*Listeria monocytogenes* ^‡^ (B)	207	72	2	149	19	3	34	0	10
*Verticillium dahliae* ^†^ (F)	215	72	15	95	12	26	67	0	17
*Acinetobacter baumannii* ^#^ (B)	191	69	0	132	6	1	52	0	6
*Toxoplasma gondii* ^‡^ (P)	154	69	3	74	6	12	57	2	5
*Streptococcus suis* ^‡^ (B)	155	63	2	118	5	0	26	4	6
*Burkholderia pseudomallei* ^‡^ (B)	100	61	0	0	0	0	0	0	4
*Candida glabrata* ^#^ (F)	207	59	0	119	13	0	74	1	3
TOTALS	13141	6111	711	5965	631	1684	3742	13141	

*Genes were mapped to the latest genome assembly and reference UniProtKB proteome where available. Symbols indicate: ^†^ plant pathogen, ^‡^ animal pathogen, ^‡†^ pathogen of both plant and animal hosts, ^#^ opportunistic pathogen usually only able to infect immunocompromised humans, ^§^ entomopathogenic fungal species used to control insect pests. Taxon indicated in parenthesis (F) fungus, (B) bacterium, (P) protozoa.

The four new most curated pathogen species are all human and/or animal infecting species, namely: *Acinetobacter baumannii*, an opportunistic bacterial pathogen that infects immunocompromised humans; *Toxoplasma gondii*, a protozoan parasite that infects most species of warm-blooded animals, including humans; *Streptococcus suis*, a major bacterial pathogen in the pig industry in tropical countries, that is also able to cause a zoonotic disease; and *Burkholderia pseudomallei*, an opportunistic bacterial pathogen that can infect humans and animals. As a result, three *Streptococcus* species with different host preferences are now present in the most annotated species list.

In total, 18 new host species are present in PHI-base in version 4.12. This includes five plant, five vertebrate and seven insect species as either the natural host(s), or the surrogate model host for testing (Supplementary Table S2). New insect test species include the cotton bollworm (*Helicoverpa armigera*), Asian malaria mosquito (*Anopheles stephensi*), American cockroach (*Periplaneta americana*), pea aphid (*Acyrthosiphon pisum*), two-spotted ladybird beetle (*Adalia bipunctata*) and the yellow fever mosquito (*Aedes aegypti*). These new host entries are mostly due to alternative non-vertebrate hosts being used instead of animal models, in line with the principles of the 3Rs (replacement, reduction, and refinement) (33). Other new hosts are curated either because of an emerging pathogenic species of increasing concern—for example, *Pseudomonas* infections on golden kiwifruit (*Actinidia chinensis*)—or because of the use of microbial biocontrol species (biopesticides) to control additional problematic hosts, such as the fungus *Metarhizium robertsii* being used to control the two mosquito species named above (Supplementary Table S2).

The high-level phenotypes (22) annotated to all PHI-base interaction entries permit taxonomically wide interspecies comparisons: these phenotype annotations are summarized for pathogen species in Table 1 and for host species in Table 2. For pathogens, the ‘reduced virulence’ phenotype has the highest number of PHI annotations at 8667 (47.7%), whereas the ‘loss of pathogenicity’ PHI phenotype has only 983 (5.4%), a split in line with previous releases (21). The ‘loss of pathogenicity’ phenotype is more frequently reported for plant infecting pathogens. The number of genes with an ‘increased virulence’ PHI phenotype when a pathogen gene is modified or deleted has more than doubled since 2019 to 969 entries. For hosts, there has been a 55% increase in the number of interactions annotated with the ‘increased virulence’ phenotype for pathogens that infect vertebrate hosts (529 interactions). With the ‘increased virulence’ category, 631 genes are from 28 of the most annotated species (Table 3). This increase emphasizes the research community's continuing efforts to identify and compare the repertoire of negative regulators in different host–pathogen systems. An ever-growing number of different protein function classes are now associated with the ‘increased virulence’ phenotype, including transcription factors, two component response regulators, various components of mitogen activated protein kinase signaling cascades, G-protein signaling components, regulators of toxin biosynthesis, and various plasma membrane transporters and secreted enzymes, particularly proteases and metalloproteases. Specifically, for bacterial pathogens, components of the type III secretion system (plant hosts only) and quorum sensing system (animal and plant hosts) are associated with increased virulence. For filamentous pathogens infecting human or animal hosts, enzymes contributing to cell wall biogenesis or integrity, or the formation of biofilms or capsules are associated with increased virulence (reviewed by (9,34)). The collected set of pathogen genes associated with increased virulence, and the accompanying sequence variation observed in hypervirulent strains, requires continual close monitoring in efforts to control disease by limiting their spread in severe local and regional occurring disease outbreaks (34).

A major curation effort for PHI-base since 2016 has been to increase coverage of pathogen effectors. An effector is an entity derived from a pathogenic or non-pathogenic species, that either activates or suppresses the host's defensive or other responses (11,35,36). The number of curated pathogen effector proteins interacting directly with one or more host species has increased by 30% since version 4.8 to 657 genes tested in 2641 interactions. Effectors now represent 14.5% of all interaction entries in PHI-base. Of these, 86% are from plant infecting pathogens and 14% are from animal and/or human infecting pathogens (Table 4). The plant pathogen data has been curated from 89 species, mostly non-cereal infecting pathogens (76 species). These plant pathogen effector entities are dominated by bacterial species and include *Ralstonia solanacearum*, which infects dicotyledonous species (and which had a 32% increase in curated effectors), various *Pseudomonas* species, and both cereal and non-cereal infecting *Xanthomonas* species. Although a wider range of hosts are now being used for *in planta* bioassays, 25% of these bioassays still use *Nicotiana benthamiana* or *Nicotiana tabacum* (352 interactions), or *Arabidopsis thaliana* (207 interactions). These three plant species are often, but not always, a non-host species for the pathogen under investigation, meaning the pathogen species is not able to cause disease on these host species even under ideal environmental conditions (36). Increasingly, effectors are reported in studies involving vertebrate hosts (primarily rodents and primates) and bacterial pathogens. For pathogens of humans and/or animals, *Salmonella enterica* has the highest percentage of effector interactions curated, but high numbers of effector interactions have also been curated for the obligate intracellular pathogen *Coxiella burnetii*, which causes the zoonotic disease Q fever in humans, and *Acinetobacter nosocomialis*, which causes nosocomial pneumonia in critically ill human patients. In studies of effectors from animal/human infecting pathogens, five non-vertebrate species, primarily *Galleria mellonella* (greater wax moth) larvae, have been used for the bioassays. For example, in *in vivo* studies involving *A. nosocomialis*, there is now an approximate 50:50 split in the use of *G. mellonella* or a rodent species for the bioassays. This again emphasizes that the international animal and human research community is gradually adopting the principles of the 3Rs.

**Table 4.** Summary of the pathogenic species providing the most information on effectors

**PLANT PATHOGENS: 58 species**	**Interactions: 2269**
**Bacteria: 16 species**	**1473**
*Ralstonia solanacearum*	787
*Xanthamonas* species	447
*Pseudomonas* species	204
*Erwinia amylovora*	15
*Burkholderia glumae*	15
**Fungus: 21 species**	**421**
*Pyrenophora tritici-repentis*	138
*Magnaporthe oryzae*	84
*Passalora fulva*	57
*Fusarium oxysporum*	30
*Verticillium dahliae*	26
*Ustilago maydis*	17
*Ustilaginoidea virens*	13
*Leptosphaeria maculans*	12
**Obligate fungal biotrophs: 5 species**	**72**
*Melampsora* species	34
*Puccinia* species	28
*Blumeria* species	10
**Protist - 10 species**	**282**
*Hyaloperonospora arabidopsidis*	127
*Phytophthora sojae*	56
*Phytophthora capsici*	40
*Phytophthora infestans*	41
**Nematodes and insects: 3 species**	**10**
**HUMAN/ANIMAL PATHOGENS: 31 species**	**Interactions: 371**
**Bacteria: 29 species**	**357**
*Salmonella enterica*	137
*Coxiella burnetii*	46
*Acinetobacter nosocomialis*	30
*Burkholderia pseudomallei*	24
*Legionella pneumophila*	23
*Yersinia* species	19
**Fungi: 1 species**	**2**
*Beauveria bassiana*	2
**Protozoan: 1 species**	**12**
*Toxoplasma gondii*	12

With ever increasing concern over climate change and its impact on global food and feed security, the international research community is being encouraged to investigate plant–pathogen interactions in crop species. The interaction entries involve major food and feed crops: namely wheat (1949), rice (1,581), maize (770), barley (522), tomato (694), potato (143) and *Brassica* species (198) providing 32% of the data in PHI-base (5857 interactions) and involve 89 pathogenic species (60% of plant pathogen species in PHI-base). The cereal interaction data dominates at 4820 entries from 43 pathogenic species that are able to cause disease on single or multiple plant tissues and organs (i.e. leaves, flowers, panicles, seeds, stem bases, roots) on one or more of these four crop species. Of these, 31 species of Ascomycete fungi, seven bacteria species and five species of Basidiomycete fungi contribute the data for 3706, 665 and 449 interactions, respectively. Cereal pathogenic species of growing economic and scientific importance globally include *Ustilaginoidea virens*, which causes false smut disease of rice; *Puccinia striiformis*, which causes yellow rust disease and stripe rust disease of wheat; and *Burkholderia glumae*, which causes bacterial seedling blight, sheath rot, panicle blight and seed rot.

### Amending strain and disease names

A pervasive problem for the curation of hosts and pathogens is the integration of strain names, as there are no existing standards for most of the species and researchers often refer to strains using varying nomenclature and abbreviations. To partially address this, we have manually reviewed and amended the pathogen and host strain names included in PHI-base version 4.12. Strain names were amended to remove typographical variation and variant (or erroneous) spellings. The primary strain name was chosen based on which name was most common in the literature curated by PHI-base or had the most occurrences in the wider pathogen–host literature. Where possible, strain names have been amended to follow the nomenclature of the relevant authority: currently, only Mouse Genome Informatics (http://www.informatics.jax.org) has been used as an authority, for strains of *Mus musculus*. Otherwise, strains were cross-referenced by querying their respective species in the Taxonomy database provided by UniProt. Other changes include prefixing all plant cultivars with ‘cv.’ and standardizing the abbreviated forms of taxonomic prefixes (e.g. ‘subsp.’). Of the 3,083 unique strain names in the database, 1075 host strains and 566 pathogen strain names were affected by these changes.

Disease names were amended to remove typographical variation and variant spellings. Human diseases were cross-referenced with the Mondo Disease Ontology (37), which merges terms from multiple disease ontologies, including the Human Disease Ontology (38), Human Phenotype Ontology (39) and the NCI Thesaurus OBO Edition (40). We were unable to locate general-purpose disease ontologies that could be used to cross-reference animal or plant diseases. Other key changes included clearly delineating disease names (where multiple diseases caused by a single pathogen are combined in one disease name), removing redundant mentions of ‘disease’, and using a consistent method for indicating the relevant host for the disease: for example, ‘rice blast’ and ‘blast disease of rice’ are both formatted as ‘blast (rice)’. In total, 351 of the 610 unique disease names in the database were affected by these changes.

### Collaboration with Ensembl Genomes

PHI-base has an active collaboration with the Ensembl Genomes resource (23) in which manually curated data from PHI-base are mapped regularly onto pathogen genes. Release 105 of Ensembl Genomes has the annotation of 302 protists, 1762 fungal and 26 837 bacterial proteins regarding their host interaction role(s) as obtained from PHI-base. These annotations can be searched using PHI-base accessions or accessed via BioMart (41). These annotations, when visualized alongside their comparative analysis data with closely related species, can help researchers form testable hypotheses for genes in comparable pathogens.

### Dissemination of PHI-base phenotypes to other databases and resource providers

PHI-base is committed to making its data reusable, and follows the FAIR data principles (18). All data in PHI-base are distributed under a Creative Commons license (Creative Commons Attribution 4.0 International Public License). PHI-base source code and data are available on GitHub repositories (see the Data Availability section). Starting with PHI-base version 4.12, the PHI-base dataset is also published in CSV format through Zenodo, a European open-access repository hosted at CERN, that automatically assigns persistent DOIs to datasets. The Pathogen Host Interaction Phenotype Ontology (PHIPO) (http://www.obofoundry.org/ontology/phipo.html), developed for PHI phenotype curation, is available through the OBO Foundry (42).

As part of the European ELIXIR ‘Data for Life’ project, PHI-base also provides data for species, genes and proteins available in the database FungiDB (25) and the UniProt Knowledgebase (UniProtKB) (27) for genome and protein annotation, respectively. FungiDB release 53 (July 2021) includes PHI-base phenotypes for 3423 proteins across 58 pathogens. In UniProtKB (release 2021_02), 5485 proteins from 522 organisms have links to PHI phenotypes. Gene Ontology (GO) curation is made available through submission to the GOA (28) and GO (43) databases and is also displayed in UniProtKB, Ensembl Genomes, FungiDB and the NCBI protein database (23,25,27,44).

### PHI-base usage

Over the last three years, users of PHI-base originated from 100 countries over six continents. During this period, the PHI-base website (www.phi-base.org) was accessed on average by 2000 users per year, with 10 searches per user. On average, the BLAST service (PHIB-BLAST) attracts more users than the PHI-base website (2,770 users per year). The PHI-base database is downloaded on average 740 times per year. To date, 550 peer reviewed publications have cited PHI-base, and over 30% of these publications have appeared since 2019. All publications citing PHI-base use are given in the ‘About us’ section of the database. Most researchers use PHI-base for the analysis of newly generated whole genome sequences and transcriptomes, and for comparative transcriptomics. These studies are published in the research areas of microbiology (26%), biotechnology (23%), biochemistry (20%), plant sciences (16%) and other more applied areas (15%) (data derived from Clarivate Web of Science™, September 2021).

### Novel use case studies

The discovery of novel virulence genes is an expensive and time-consuming process. Frequently, these genes are characterized by highly diverse sequences. Since 2005, advances in machine learning (ML) approaches and biological understanding have enabled the development and application of ML algorithms for the discovery of bacterial virulence factors (45). The increase in PHI-base data opened up the possibility to apply similar approaches for eukaryotic pathogens. Most recently, PHI-base data were included in ML approaches used for the prediction of fungal and oomycete pathogen effectors, resulting in the development of online prediction tools, such as EffectorP (http://effectorp.csiro.au/) (46,47). Kristianingsih and MacLean (48) found that small ML training sets can be used to inform highly accurate effector gene predictions.

Molecular interactions featuring proteins in PHI-base are another increasingly investigated topic by PHI-base users. Discovering the functional interactions of pathogen and host proteins is considered to be a good route to foster the discovery of novel intervention targets for controlling pathogens (30). Disrupting critical protein–protein interactions (PPIs) can be an important approach in the development of new anti-infectives of medical importance (49). Similar approaches are being investigated to control plant pathogens (50). Although there are currently only a small number of PPI datasets available for most pathogens and their hosts, increasingly large data sets have become available for model species such as baker's yeast (*Saccharomyces cerevisiae*), fission yeast (*Schizosaccharomyces pombe*), roundworm (*Caenorhabditis elegans*), fruit fly (*Drosophila melanogaster*), zebrafish (*Danio rerio*) and the house mouse (*Mus musculus*) (51). These model datasets allow construction of biological networks linking together the biological entities that are implicated in physical interactions (e.g. PPIs, enzyme binding to a substrate), or are shown to be associated by co-expression and/or co-localization. For PHI-base pathogen and host species, insufficient experimental data is available to construct similar networks. Other authors have used various computational methods to overcome a similar lack of data: these methods include an interolog approach that relies on sequence similarity between proteins from different species; identification of conserved Pfam molecule binding domains in PHI-base proteins to identify interactors; and generation of network-extracted ontologies to annotate transcriptomics data (52,53). These methods were used by three recent studies that specifically took the high-level phenotype annotations assigned to PHI-base proteins to construct networks of rice-pathogen interactions (54), to identify and build annotated networks for putative virulence factors for 14 Ascomycete fungal pathogens (55), and to generate ontologies, extracted from an interaction network, that led to the identification of the PEP8 protein in the human infecting fungal pathogen *Candida albicans*. PEP8 is likely involved in retrograde vesicle transport, with a function in hyphal development and immune evasion (56).

### Current work and future plans

**Figure 1.**
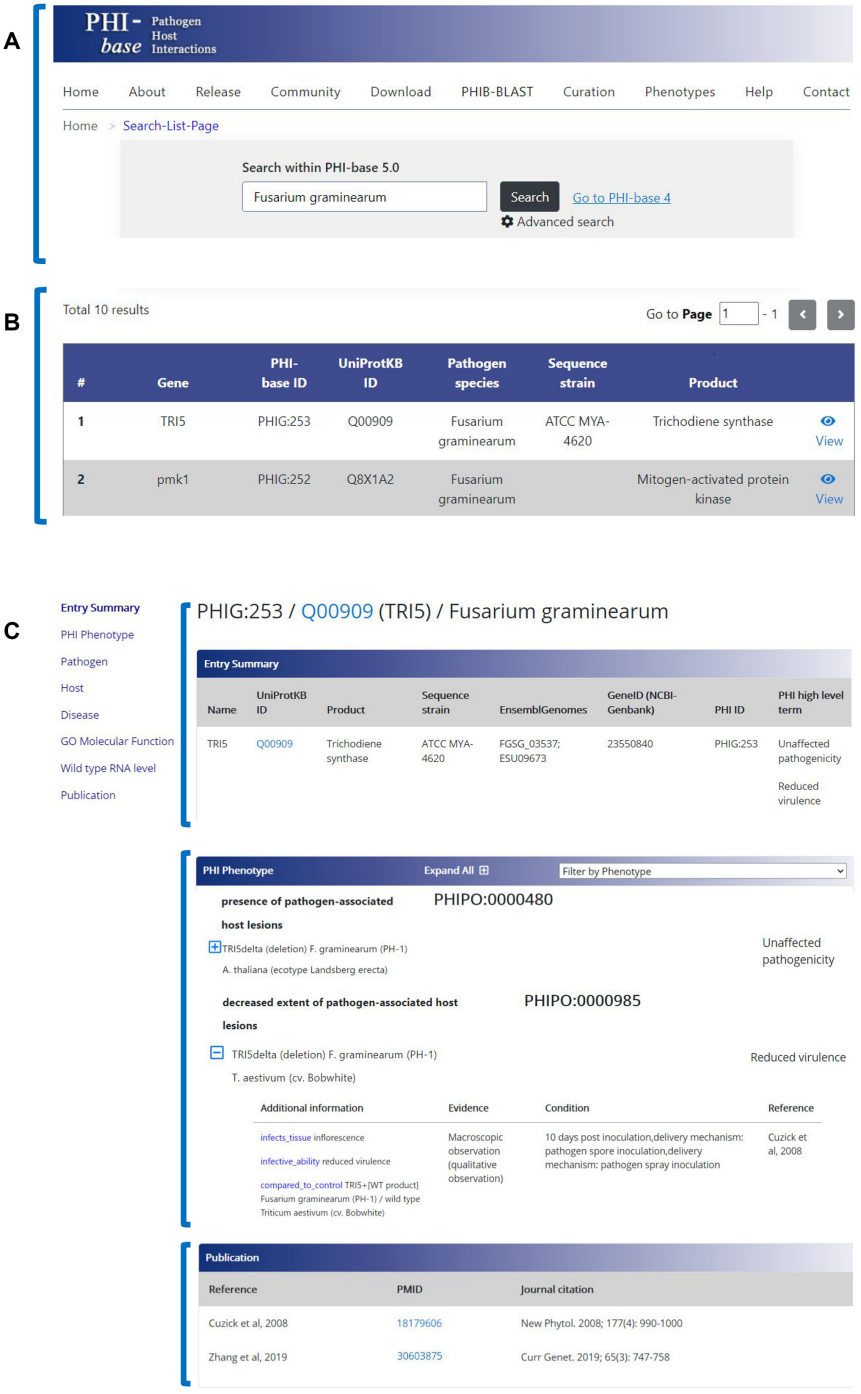
An example of a PHI-base 5 gene centric web page for the aggregated display of all relevant peer reviewed articles curated using the community curation tool, PHI-Canto. (**A**) The PHI-base 5 home page provides search functionality with autocomplete, links to contact and other information as well as a link to the current article centric version 4 of PHI-base. (**B**) Search results for the fungal plant pathogen ‘*Fusarium graminearum*’ retrieve 10 genes available for this species (only two genes shown). The ‘View’ button on the far right allows users to retrieve information on specific genes, e.g. *TRI5* or *pmk1*. (**C**) Results retrieved for the *TRI5* gene. The sidebar (left) allows users to jump to any of the eight specific record sections. The selected ‘Entry Summary’ field (in bold) provides gene information including the assigned stable PHI gene identifier (PHIG:) and a link to UniProtKB. Another selected field ‘PHI Phenotype’ lists the details of different host, pathogen, interaction, and phenotypes using terms from the PHIPO ontology. Also included in the ‘PHI Phenotype’ field is the assigned high-level phenotype ‘reduced virulence’ or ‘unaffected pathogenicity’ for the gene deletion mutant *TRI5delta* tested on infected hosts wheat (*T. aestivum*) or Arabidopsis (*A. thaliana*), respectivel*y*. The ‘Publication’ field lists all references used for the curation of the gene. Note: for users wishing to browse the entire database, add a single asterisk (*) into the search box (Panel A).

We are developing a new user interface for the PHI-base database (PHI-base 5) (phi5.phi-base.org). The PHI-base 5 website provides a gene-centric view of the data. The aggregated data is presented on a single page corresponding to the gene in a single species (Figure 1). This contrasts with PHI-base 4, where the pathogen–host interaction is the central concept, the gene only exists as part of the interaction, and no gene-focused view is provided. Development of PHI-base 5 was prompted by two requirements. First, PHI-base users requested PHI phenotype information to be displayed in association with a gene (or its protein). Second, a new user interface is required to display the additional data types curated by authors using our multi-species community curation tool, PHI-Canto (21), which is based on the Canto tool developed by PomBase (57). When using the curation tool, the gene's molecular function and expression level is captured independently from the phenotype annotations. PHI-Canto can be used by researchers to curate and submit their own published pathogen–host datasets. Submitted curation will be reviewed by species experts and included in PHI-base 5, providing an additional mechanism for data providers to satisfy funding requirements to make published research data electronically available. PHI-Canto is currently used for curation by the PHI-base team, but we plan to trial community curation with the plant and medical research communities over the next 6–9 months.

The first online version of PHI-base 5 contains curated data from 26 publications, covering 18 pathogens and providing 873 annotations, curated using PHI-Canto (Supplementary Table S3). During the next 12 months, the plan is to migrate all 18 190 PHI phenotypes currently only available in PHI-base 4 to the new PHI-base 5 gene-centric display. This data migration process will require extensive manual review and possibly retroactive curation, since the schemas of the two database versions are not compatible: PHI-base 5 has support for many more data types and annotation types compared to PHI-base 4, and some data types are curated in different ways or in different formats in PHI-base 5. After the data is migrated, we plan to retain an archived version of the PHI-base 4 website on the phi-base.org domain until 2026.

To further improve findability on the web, we plan to include Schema.org markup (www.schema.org) on our gene-centric PHI-base 5 pages: this markup will enable structured data to be extracted from the gene pages by semantic search engines, and therefore allow those search engines to understand the meaning of the page. Version 13.0 of Schema.org (released July 2021) adds terms from the Bioschemas community (https://bioschemas.org) which cover multiple concepts also modelled in PHI-base records, such as genes, proteins, taxonomic ranks, and molecular entities (chemical compounds).

Knowledge graphs provide additional data tools to investigate large-scale datasets. To enhance the querying and display of PHI-base data we plan to build multi-species pathogen-host gene networks jointly with KnetMiner (58). KnetMiner provides researchers with integrated data that connect genetic, omics and phenotypic information from a wide range of public databases. These networks will permit querying both for pathogen and host genes, and the multiple data types curated in PHI-base.

Ensembl Genomes are developing a data model to store protein–protein interactions identified in PHI-base, linking pathogen effectors to their first host targets. These will be stored in a new resource to be available on the gene pages (both for hosts and pathogens) and via direct downloads of the data. Given the wide representation of species within Ensembl (vertebrates to metazoa to plants) (56), this will provide a platform that can capture relationships between any two proteins from any two species, thus greatly expanding the potential scope of this resource to many fields of study, such as agriculture, human and animal health, and ecology.

## DATA AVAILABILITY

1. PHI-base 4: www.phi-base.org
2. PHIB-BLAST: phi-blast.phi-base.org
3. GitHub: github.com/PHI-base
4. PHI-Canto: canto.phi-base.org
5. PHI-base 5: phi5.phi-base.org
6. Ensembl Genomes: ensemblgenomes.org
7. FungiDB: fungidb.org
8. KnetMiner: knetminer.com
9. UniProtKB: www.uniprot.org
10. Zenodo: zenodo.org

## Supplementary Material

gkab1037_Supplemental_File
